# Volitional Control of Neuromagnetic Coherence

**DOI:** 10.3389/fnins.2012.00189

**Published:** 2012-12-26

**Authors:** Matthew D. Sacchet, Jürgen Mellinger, Ranganatha Sitaram, Christoph Braun, Niels Birbaumer, Eberhard Fetz

**Affiliations:** ^1^Neurosciences Program, Stanford University School of MedicineStanford, CA, USA; ^2^Department of Psychology, Stanford UniversityStanford, CA, USA; ^3^Institute of Medical Psychology and Behavioral Neurobiology, University of TübingenTübingen, Germany; ^4^Department of Biomedical Engineering, University of FloridaGainesville, FL, USA; ^5^Sri Chitra Tirunal Institute of Medical Sciences and TechnologyTrivandrum, India; ^6^Magnetoencephalography-Center, University of Tübingen Medical SchoolTübingen, Germany; ^7^Centro Interdipartimentale Mente – Cervello Center for Mind/Brain Sciences, University of TrentoTrento, Italy; ^8^Department of Psychology and Cognitive Science, University of TrentoTrento, Italy; ^9^San Camillo Hospital, Scientific Institute for Research, Hospitalization and Health CareVenice, Italy; ^10^Department of Physiology and Biophysics, University of WashingtonSeattle, WA, USA

**Keywords:** neurofeedback, magnetoencephalography, phase synchronization, functional neuorimaging, methods

## Abstract

Coherence of neural activity between circumscribed brain regions has been implicated as an indicator of intracerebral communication in various cognitive processes. While neural activity can be volitionally controlled with neurofeedback, the volitional control of coherence has not yet been explored. Learned volitional control of coherence could elucidate mechanisms of associations between cortical areas and its cognitive correlates and may have clinical implications. Neural coherence may also provide a signal for brain-computer interfaces (BCI). In the present study we used the Weighted Overlapping Segment Averaging method to assess coherence between bilateral magnetoencephalograph sensors during voluntary digit movement as a basis for BCI control. Participants controlled an onscreen cursor, with a success rate of 124 of 180 (68.9%, sign-test *p* < 0.001) and 84 out of 100 (84%, sign-test *p* < 0.001). The present findings suggest that neural coherence may be volitionally controlled and may have specific behavioral correlates.

## Introduction

Coherence and synchrony between different brain regions reflect neural interactions and information exchange between the regions (Siegel et al., 2012). An abundance of research suggests the prevalence of large-scale coherence of neural signals between disparate brain regions during various cognitive processes. For example, one study of local field potentials in monkeys found “top-down” attentional modulation accompanied by increased low-frequency fronto-parietal synchrony, and “bottom-up” attentional control with stronger higher-frequency synchrony in the same regions (Buschman and Miller, 2007). In humans, large-scale coherence of neural signals has been observed using magnetoencephalography (MEG) and electroencephalography (EEG); for example, in top-down modulation of attentional goals and subsequent working memory performance (Zanto et al., 2011), top-down visual attention allocation (Phillips and Takeda, 2009), and associative learning (Miltner et al., 1999).

Since coherence indicates intracortical communication and functional connectivity (Siegel et al., 2012), increasing or decreasing coherence should have behavioral and cognitive correlates. Volitional control of coherence could be directly investigated through real-time neurofeedback, and could be implemented in brain-computer interfaces (BCI) that allow for human-machine communication and machine control using brain activity. A learned change in neural coherence may change a particular type of behavior dependent upon coherent activities.

To our knowledge, studies to date have not yet explored the feasibility of volitional control of coherence. In this proof-of-concept study, we explored whether coherence between bilateral MEG sensors could be controlled in two participants. During parameter selection, participants were asked to perform a digit extension-contraction task in order to identify the magnetic field sensors that are most associated with the modulation of sensorimotor μ rhythm (SMR; Mellinger et al., 2007). Then behaviors that altered coherence were identified (five different behaviors were compared: left index finger tapping, right index finger tapping, bimanual synchronous index finger tapping, bimanual alternative index finger tapping, and rest). Subsequently, feedback testing sessions were conducted, during which on-line feedback was provided on the current level of coherence using the sensors, frequencies, and behaviors previously identified. During these sessions, participants were instructed to perform one of two behaviors to control movement of a cursor toward a target on a screen. Finally, off-line analyses were completed to assess possible confounds, including whole-head movement, single-source signal propagation, muscle artifacts, artifacts from single-trial analysis, power domination of the coherence signals, and stability of coherence differences. Our results eliminated these possible confounds, indicating that coherence was successfully controlled.

## Materials and Methods

### Participants

Two healthy adult volunteers participated in the study (both male and right handed, 23 and 24 years old). Participants gave informed consent and the University of Tubingen ethics committee approved this study.

### Materials

A MEG (CTF Inc., Vancouver, BC, Canada) at the MEG-Center at the University Clinic of Tübingen was used (shielded room, VacuumSchmelze, Hanau, Germany; 275 axial gradiometers with a baseline of 5 cm, 586 Hz sampling rate, anti-aliasing filter at 208 Hz, continuous head localization). Participants sat upright watching a 40 cm × 30 cm screen located at eye level. A portion of the screen (20 cm × 15 cm) was used in order to reduce head movements. Recordings lasted approximately 3 h for each participant, and comprised of two recording sessions on separate days. Each participant completed three types of sessions: sessions used to identify sensors of interest (parameter selection), sessions used to identify behaviors of interest, and neurofeedback sessions. Only during the neurofeedback sessions did the participants receive visual feedback about the strength of coherence. The BCI2000 (Schalk et al., 2004; bci2000.org) was used for real-time analysis and display during recording sessions. Off-line analyses were conducted using MATLAB (The Mathworks, Inc.) with in-house scripts.

### Coherence measurement

To measure coherence between neural signals, we used a standard method which employs correlation between a time series of short-term Fourier coefficients. For a pair of signals *x* and *y*, coherence *C_xy_* is a function of frequency *f*, and is given as the squared correlation coefficient between their associated time series of Fourier coefficients $\tilde{x}\left(f\right)$ and ${ỹ}_{i}\left(f\right)$:

$$C_{xy}\left(f\right)=\frac{\left|\underset{i}{\sum }{\tilde{x}}_{i}\left(f\right){ỹ}_{i}^{*}\left(f\right)\right|}{{\underset{j}{\sum }\left|{\tilde{x}}_{j}\left(f\right)\right|}^{2}.{\underset{k}{\sum }\left|{ỹ}_{k}\left(f\right)\right|}^{2}}$$

For each index *i*, ${\tilde{x}}_{i}\left(f\right)$ and ${ỹ}_{i}\left(f\right)$ are computed over a finite-length time window applied to both *x*(*t*) and *y*(*t*). That time window moves forward in time as *i* increases. Typically, *x* and *y* are discrete time series, and their short-term Fourier coefficients are computed by means of Discrete Fourier Transformation over short time windows, which are either rectangular windows, or involve a sidelobe suppression window function such as a Hamming (used in the current study), Hann, or Blackman window. Window width determines resolution in the frequency domain; by overlapping windows in time, the time series of Fourier coefficients may have improved estimation quality. This coherence estimation algorithm is widely used, e.g., in Matlab’s implementation of the mscohere() function. In the current study we use the term Weighted Overlapping Segment Averaging (WOSA) to signify this method of coherence estimation.

In our data analysis, coherence was determined from discrete Fourier coefficients computed over a moving window with a length of 208 ms, and an overlap of 50%. Determined by the window length a frequency resolution of 4.81 Hz was obtained. During off-line analysis, an FFT-based method was used to calculate the full coherence spectrum (0–586 Hz); for each trial 8 s periods were analyzed. For computation of on-line feedback, an FIR-based method was used to estimate coherence only at individual frequencies that had been chosen based on off-line analysis. Data were taken from a buffer that contained the signals of the previous 3 s.

### Identifying parameters for feedback control

The SMR was selected as the signal of interest because it occurs with characteristic spatial distribution over bilateral sensorimotor cortex (Kuhlman, 1978), therefore constraining channel selection during BCI parameter identification. Additionally, because bodily movement modulates SMR (Babiloni et al., 1999), it was hypothesized that movement (in the current study: finger tapping) may also modulate bilateral coherence measured from sensors associated with the SMR.

A paradigm used in Mellinger et al. (2007) to identify those MEG channels most associated with SMR will be briefly described here. Participants performed repetitive, self-paced, finger extension-contraction movements interspersed with rest periods. Subjects’ hands were palm-up on separate armrests. The task was timed by visual cues that appeared for 5 s, with 2 s intervals between cues. Subjects were instructed to conduct the movements only during the visual cue period. To minimize potential effects from initiating and terminating movement, the middle 3 s periods (1 s buffer on the ends) were included in analysis. Data from this task was analyzed off-line by computation of topographical and spectral maps of determination coefficients (squared correlation values) of power between left hand and right hand movements. These maps allow for assessment of the amplitude variance accounted for by the task condition (i.e., left hand movements, right hand movements, or rest) at a given frequency. From this map, a single frequency band was used to identify the sensors with the highest correlation values in the target positions, taking into account the characteristic spatial localization associated with the SMR. For spatial filtering, sensors were linearly combined with weights of +1 or −1 according to the relative polarity of the magnetic signal at different sensor locations (influx and outflux of the magnetic field). This paradigm permits characterization of a set of sensors associated with a participant’s SMR for subsequent coherence feedback.

We used an additional recording paradigm to identify behaviors for which strongest interhemispheric coherence differences were elicited and that appeared to be suitable for the subsequent testing of coherence feedback. In this paradigm participants were instructed to perform five different behaviors: left index finger tapping, right index finger tapping, bimanual synchronous tapping, bimanual alternative tapping, and rest. The tapping behaviors were self-paced. Behaviors were performed for 5 s intervals with 1–3 s intervals between trials. Each run consisted of 20 trials (i.e., four for each behavior). Off-line analyses (using regression analyses of WOSA coherence estimates at the sensors previously identified) were conducted to assess which pairs of behavior elicited the largest difference in coherence, and at what frequency the greatest difference occurred. Of note, the SMR frequency used to identify sensors of interest was not used to constrain the frequencies analyzed to assess the largest difference in coherence between behaviors (statistical significance was corrected for multiple comparisons with the Bonferroni method). The behavior pairs that were finally identified would correspond to the behaviors to be initiated during on-line feedback training runs.

### Feedback

**Figure 1:**
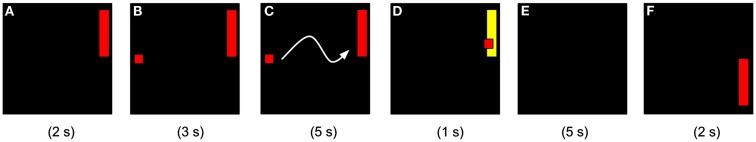
**Schematic of feedback trials**. **(A)** Target appears for 2 s (indicating goal of current trial); **(B)** cursor appears for 3 s while real-time coherence is calculated; **(C)** for 5 s the cursor moves in the *x* direction at a constant speed and *y* velocity is determined by comparison against the average of the last three trials of each type (six trials total); **(D)** if hit, the target changes to yellow for 1 s; if missed, the target remains red; **(E)** inter-trial interval of 5 s; **(F)** a new trial begins.

A single feedback run consisted of 20 trials. In each trial a target appeared at the right spanning half of the vertical length of the screen, either on the top-right or bottom-right side. This target cued the participant to either increase or decrease coherence, when the target was in the upper portion of the screen and lower portion of the screen, respectively. The location of the target indicated to the participants which behavior to conduct from the two behaviors previously identified to increase and decrease coherence; the behavior was to be performed when the target appeared. After 2 s a cursor appeared on the left side of the screen. Three seconds later the cursor moved toward the right side of the screen at a constant velocity in the *x* direction, *v_x_*, and a velocity in the *y* direction, *v_y_*, that was dependent on the value of coherence being measured. Figure 1 shows a schematic of a feedback trial. A linear function described *v_y_* in terms of amplitude of the coherence measure: *v_y_* = *b*(*S − a*). Where intercept *a* and gain *b* were adapted dynamically to optimize control over the cursor’s movement (McFarland et al., 1997):

$$\begin{array}{rcll} a & =\frac{1}{2}\left({\bar{S}}_{\text{top}}-{\bar{S}}_{\text{bottom}}\right) & & \\ \frac{1}{b} & \propto \left({\bar{S}}_{\text{top}}-{\bar{S}}_{\text{bottom}}\right) & & \end{array}$$

Where ${\bar{S}}_{\text{top}}$ and ${\bar{S}}_{\text{bottom}}$ are adaptive on-line estimates of class means, trials in which the target appeared on top and indicated that the behavior that increased coherence was to be performed, and trials in which the target appeared at the bottom and thus indicated that the behavior should decrease coherence, respectively. The adaptive class means were computed over the last three trials of the given type (i.e., increase or decrease trials). In each trial of feedback training the cursor moved to the right side of the screen during a total of 5 s. If the target was hit, it changed from red to yellow and maintained its color for 1 s; if it was not hit, it remained red for 1 s. An interval of 5 s occurred between each trial. Participants completed different numbers of feedback runs (subject 1 completed nine runs, and subject 2 completed five runs). Each participant completed one or more training runs before feedback testing. In order to reduce computational cost, WOSA coherence was not calculated for all frequencies. Instead, a FIR filter was used to extract signals at the frequency of interest and subsequent analyses were conducted on these signals. Of note, the frequency of interest used in feedback training was slightly altered from the frequency identified during parameter identification because the on-line feedback system required integer values for window length for the FIR filter, which was not required for off-line analyses which analyzed the entire spectrum (0–586 Hz).

### Off-line analyses to control for confounds

Regression analysis of power by condition across frequencies by channels was conducted to assess muscle artifacts. This data was also topographically explored for neck, eye, and head movements near the frequency of interest. To eliminate the possibility that single-trial analysis might introduce the effects of BCI control, all trials were concatenated and coherence was analyzed and compared to the single-trial analysis. Analysis of power at single sensors was conducted to control for the possibility that power was driving the BCI. To assess variation in run-by-run coherence, coherence in each run was analyzed independently.

## Results

### Subject 1

**Figure 2:**
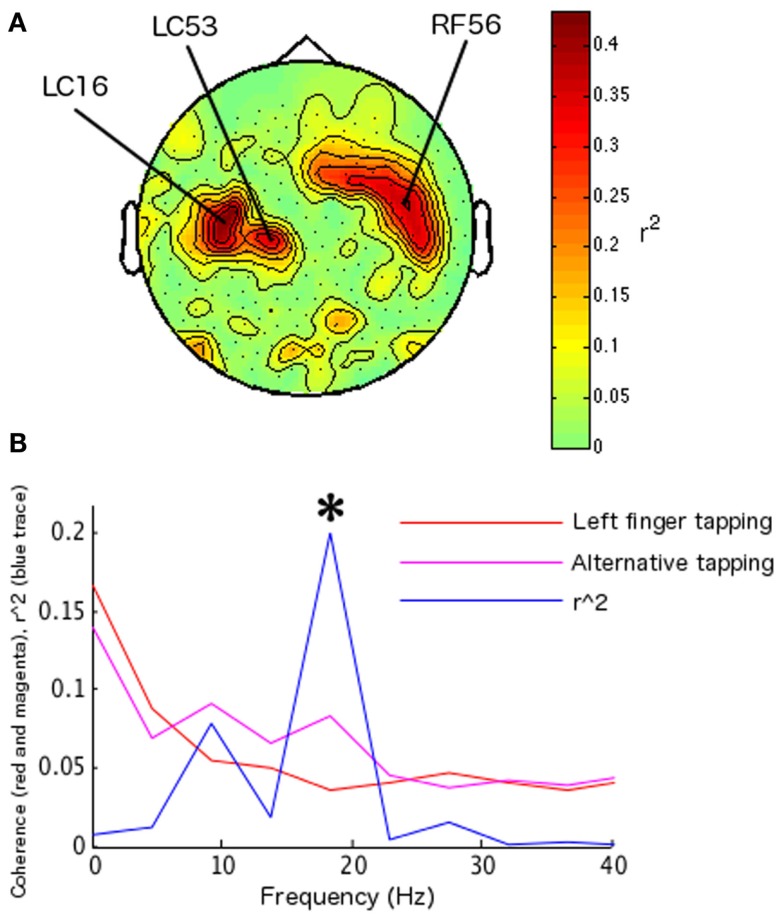
**Parameter identification for subject 1, including channels, behaviors, and frequency of interest**. Spectral map of determination coefficients (*r*^2^ values) between left and right hand movements (as in Mellinger et al., 2007) across frequencies and channels were used to identify an SMR-related frequency. **(A)** Topographical map of determination coefficients between left hand versus right hand movements at the frequency used to identify SMR-related sensors; this was the frequency of interest during coherence feedback for this participant. The location of extreme determination coefficients is in accord with spatial localization of the SMR, that is, over sensorimotor cortex (Kuhlman, 1978). **(B)** Plot of the largest regression values for subject 1 during behavior identification, indicating the frequencies at which the difference in coherence between the behaviors of interest was largest. Left index finger tapping versus bimanual alternate tapping elicited the largest determination coefficient (determination coefficients for other behavior pairs not shown). These behaviors were used to control real-time coherence. Asterisks indicate statistical significance at frequencies of interest for on-line feedback.

During the identification of parameters for feedback training, two sensors were identified in the left hemisphere (LC16, LF53 reversed sign), and one in the right hemisphere (RF56; Figure 2A) at 22.5 Hz (presumably a harmonic of the SMR, given its localization and additional peak at ∼9 Hz). Left index finger tapping was optimally differentiated from bimanual alternative tapping at 18.31 Hz (surviving Bonferroni correction across 12 frequency bins in 10 paired conditions; *r*^2^ = 0.2010, df = 78, *t* = 4.430, *p* < 0.0001; Figure 2B). During feedback, the frequency of interest and the maximal differentiable behaviors were utilized, while a set of new sensors associated with the SMR were identified. These sensors were as follows: four sensors were identified on the left hemisphere (LF24, LF25, and reversed sign LF46 and LF54); five sensors were identified on the right hemisphere (RF54, RF55, RF51, and reversed sign RT23 and RT26). The FIR filter was set at central frequencies of 19.23 and 24.085 Hz (the two closest allowable frequencies to 22.5 Hz compatible with the on-line system). During feedback, subject 1 completed 124 hits from a total of 180 trials (68.89%, sign-test *p* < 0.001; Figure 4A). Off-line analyses confirmed that the subject was increasing coherence when directing the cursor to the upper-right target and decreasing coherence when directing the cursor toward the lower-right target (at 18.31 Hz, *r*^2^ = 0.05351, df = 178, *t* = 3.1723, *p* < 0.005; at 22.89 Hz *r*^2^ = 0.0559, df = 178, *t* = 3.2464, *p* < 0.005; 18.31 and 22.89 Hz were the closest frequency bins greater and less than the on-line feedback frequency, 22.5 Hz; Figure 4B).

### Subject 2

**Figure 3:**
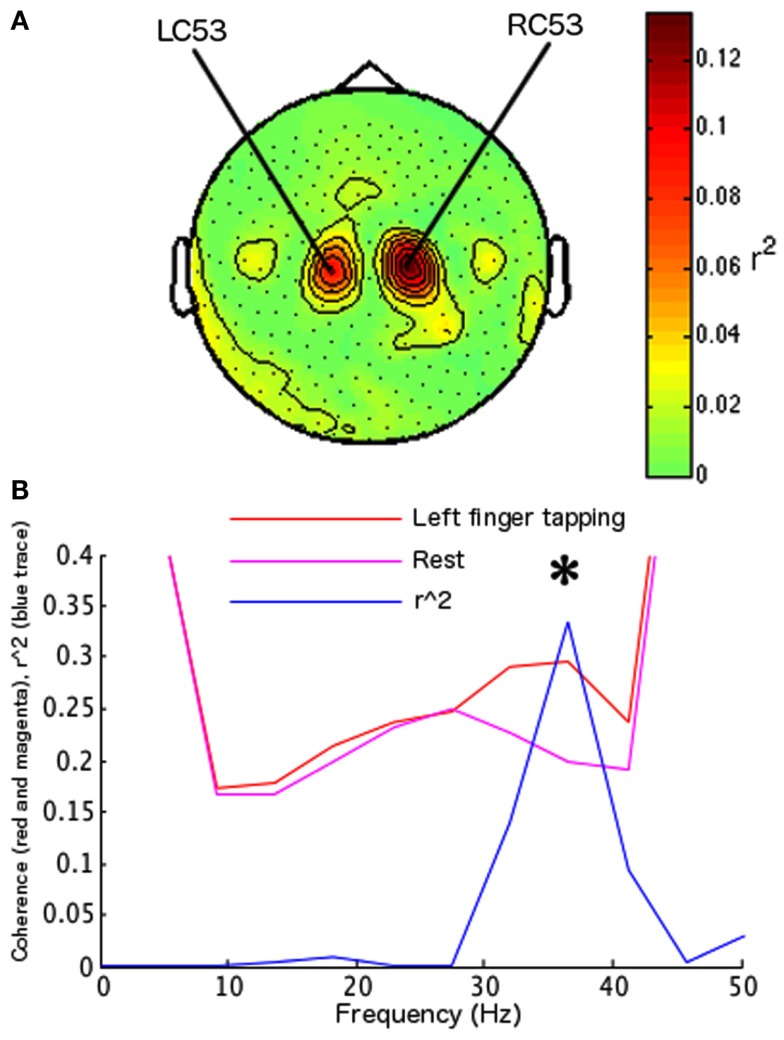
**Parameter identification for subject 2**. Same convention as Figure 2. **(A)** Topographic map of determination coefficients between left hand versus right hand movements at the frequency used to identify sensors related to the SMR. These sensors were used during behavior identification and during feedback testing. **(B)** Plot of the largest regression values for subject 2 during behavior identification, indicating the frequencies at which the difference in coherence between the behaviors of interest was largest. Left index finger tapping versus rest elicited the largest determination coefficient (determination coefficients for other behavior pairs not shown). The frequency with largest determination coefficients was used in feedback testing for this subject, and the identified behaviors were used to control coherence.

**Figure 4:**
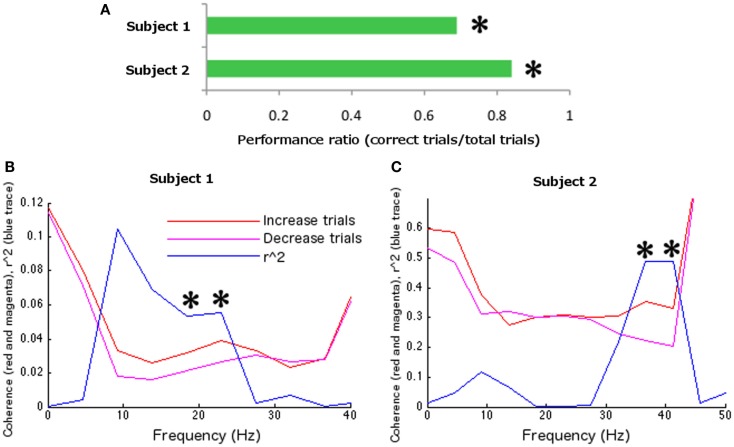
**Feedback data**. **(A)** Overall feedback performance. Both participants were able to control neural coherence. **(B)** Subject 1 and **(C)** Subject 2 coherence and corresponding determination coefficients during feedback testing between behavioral conditions across frequency (computed off-line). Asterisks indicate statistical significance at frequencies of interest, nearest to the feedback test frequency.

Using the parameter identification paradigm, one channel was selected in the right hemisphere (RC53) and one in the left hemisphere (LC53; Figure 3A) at 13.5 Hz. The largest differentiation of coherence between two behaviors occurred at 36.62 Hz (surviving Bonferroni correction across 12 frequency bins in 10 paired conditions; *r*^2^ = 0.3355, df = 78, *t* = 6.2755, *p* < 0.0001) with left index finger tapping increasing coherence relative to the rest condition (Figure 3B). Frequency for the on-line FIR filter was set to 38.46 Hz (the nearest allowable frequency for the on-line system to 36.62 Hz). During feedback testing 84 hits were recorded out of 100 trials (84%, sign-test *p* < 0.001; Figure 4A). Off-line analysis confirmed that the subject was increasing coherence when directing the cursor to the upper-right target and decreasing coherence when directing the cursor toward the lower-right target (at 36.62 Hz, *r*^2^ = 0.4903, df = 98, *t* = 9.7093, *p* < 0.001; at 41.20 Hz, *r*^2^ = 0.4868, df = 98, *t* = 9.6415, *p* < 0.001; 36.62 and 41.20 Hz were the closest frequency bins greater and less than the feedback tested frequency, 38.46 Hz; Figure 4C).

### Assessing potential confounds

Regression analysis of power by condition across frequencies and channels did not reveal signs of muscle artifact in either subject (e.g., strong *r*^2^ values spanning many frequencies across a subset of channels suggest muscle artifact; such *r*^2^ values were not found). Sensor maps of regression values did not reveal signs of neck or eye artifacts. That is, high *r*^2^ values were not observed in unexpected locations at the frequency of interest (e.g., in posterior location or anterior locations, indicating neck or eye movements respectively). High *r*^2^ values were also not observed across the entire brain, which would indicate whole-head movement corresponding to condition (increase or decrease coherence). Conducting coherence analysis on concatenated trials of each type revealed the expected pattern of coherence at the frequency of interest: trials in which the subject was to increase coherence revealed higher coherence than trials in which the subject was to decrease coherence. In subject 1, analysis of power at the frequency of interest at single channels from left and right cortex revealed that power in trials in which coherence was to increase was less than power during trials in which coherence was to decrease. In subject 2 similar analyses revealed weak power values at each right and left sensor. Coherence analyses of each run independently revealed that the coherence pattern was stable, with eight of nine trials for subject 1 (88.89%, sign-test *p* < 0.02) and five of five trials for subject 2 (100%, sign-test *p* < 0.04) showing absolute higher coherence (from 18 to 24 Hz for subject 1 and from 34 to 40 Hz for subject 2) during trials in which the subject was to increase coherence relative to trials in which the subject was to decrease coherence at the frequencies of interest.

## Discussion

We demonstrate that neural coherence as measured by MEG can be volitionally controlled in real-time. Two healthy individuals participated in our experiments involving parameter identification, and subsequent on-line testing of volitional control of coherence, and each showed significant control over the coherence signals. Off-line analyses to assess for confounds did not reveal evidence that the BCI control was caused by muscle artifacts, head/neck/eye movements, single-trial analysis, or power domination of the signal. Notably, the coherence effects were stable across runs.

It is important to clarify several methodological issues. Firstly, the feedback signal was delayed by approximately 3 s in order to accumulate enough data for a stable estimate of the coherence spectra and phases of the signals originating from multiple sensors. One may argue that such a delay could impede learning; however, previous studies training the blood oxygen level dependent (BOLD) response with fMRI and functional Near Infrared Spectroscopy (fNIRS) have shown that even several seconds of feedback delay do not adversely affect learning if the delay remains constant (Sitaram et al., 2008, 2009) as the brain implicitly takes into account the delay between response and reward to adapt its performance (Caria et al., 2011).

Secondly, an open question concerns the appropriateness of the specific interaction quantification used, i.e., WOSA coherence. An alternate technique for assessing the synchronization of spatially separated neural signals is phase synchronization (e.g., phase locking value). Phase synchronization is similar in nature to WOSA coherence but separates phase from amplitude components (Lachaux et al., 1999). Arguably, a measurement such as phase synchronization may offer more precise identification of phase information as amplitude components are discarded. However, there is the imminent possibility that the phase synchronization might be confounded by signal power due to embedded noise in the signal (Muthukumaraswamy and Singh, 2011). Thus, it is currently unclear which methods may be most appropriate for neural signal interaction based neurofeedback and BCI applications, and a comparative study might help to clarify this issue.

Our study provides a proof of the concept that MEG coherence feedback can allow participants to increase and decrease long-distance neural coherence through appropriate movements. Future protocols could target coherence measures that do not rely on finger tapping or other movements (e.g., motor or sensory imagery, or other cognitive activity), but instead rely on mental imagery guided by the feedback itself, and the brain’s intrinsic ability to learn by instrumental conditioning. Additional issues for future work include: isolating sensors of interest based on maximum coherence produced during SMR identification, using spatial filters in real-time to extract activity from source space and utilizing oscillations other than the SMR rhythm (e.g., signals from prefrontal cortex). A significant question concerns the cognitive strategies used to control interaction between particular cortical regions.

Self-regulation of coherence between different cortical locations in the various frequency ranges of MEG and EEG could be used to establish a causal link between dynamic intracortical connectivity involved in perception, cognition, and behavior. Learned self-reproduction of coherence producing a particular perceptual, cognitive, or behavioral process allows for the assessment of the causal consequences of the learned coherence. Of note, such a neuroscientific protocol exchanges the conventional relationship between independent and dependent variables to a relationship in which the brain activity is the independent variable and behavior is the dependent variable (Weiskopf et al., 2007; Caria et al., 2011). Additionally, measurements conducted on a session-by-session, run-by-run, or trial-by-trial basis can allow for the quantification of learning effects due to feedback training.

Many neuropsychiatric and neurological disorders show pathological changes in long-range coherence/synchrony. A vast literature has documented such changes in a variety of diseases, including schizophrenia (Uhlhaas and Singer, 2010), unipolar major depressive disorder (Knott et al., 2001), autism (Murias et al., 2007; Barttfeld et al., 2011), and attention-deficit/hyperactivity disorder (Barry et al., 2011). Disruption of coherent neural oscillations has also been observed in movement disorders such as Parkinson’s disease (Stoffers et al., 2008) and neurodegenerative pathologies including Alzheimer’s disease (Yener and Basar, 2010). Recently, real-time fMRI neurofeedback training has demonstrated changes in the effective connectivity of the brain network and consequent behavior (Rota et al., 2010; Lee et al., 2011; Ruiz et al., 2011). Coherence-based neurofeedback training may promote the prevention, rehabilitation, and control of symptomatology in these and other psychiatric, developmental, and neurological disorders.

## Conflict of Interest Statement

The authors declare that the research was conducted in the absence of any commercial or financial relationships that could be construed as a potential conflict of interest.
